# Changes in structural network topology correlate with severity of hallucinatory behavior in Parkinson’s disease

**DOI:** 10.1162/netn_a_00078

**Published:** 2019-03-01

**Authors:** Julie M. Hall, Claire O’Callaghan, Alana J. Muller, Kaylena A. Ehgoetz Martens, Joseph R. Phillips, Ahmed A. Moustafa, Simon J. G. Lewis, James M. Shine

**Affiliations:** School of Social Sciences and Psychology, Western Sydney University, Milperra, NSW, Australia; Brain and Mind Centre, University of Sydney, Camperdown, NSW, Australia; Brain and Mind Centre, University of Sydney, Camperdown, NSW, Australia; Department of Psychiatry and Behavioural and Clinical Neuroscience Institute, University of Cambridge, Cambridge, United Kingdom; Brain and Mind Centre, University of Sydney, Camperdown, NSW, Australia; Brain and Mind Centre, University of Sydney, Camperdown, NSW, Australia; School of Social Sciences and Psychology, Western Sydney University, Milperra, NSW, Australia; Brain and Mind Centre, University of Sydney, Camperdown, NSW, Australia; School of Social Sciences and Psychology, Western Sydney University, Milperra, NSW, Australia; MARCS Institute, Western Sydney University, Milperra, NSW, Australia; Brain and Mind Centre, University of Sydney, Camperdown, NSW, Australia; Brain and Mind Centre, University of Sydney, Camperdown, NSW, Australia

**Keywords:** Parkinson’s disease, Visual hallucinations, Diffusion tensor imaging, Network topology, Graph theory, Connectomics

## Abstract

Inefficient integration between bottom-up visual input and higher order visual processing regions is implicated in visual hallucinations in Parkinson’s disease (PD). Here, we investigated white matter contributions to this perceptual imbalance hypothesis. Twenty-nine PD patients were assessed for hallucinatory behavior. Hallucination severity was correlated to connectivity strength of the network using the network-based statistic approach. The results showed that hallucination severity was associated with reduced connectivity within a subnetwork that included the majority of the diverse club. This network showed overall greater between-module scores compared with nodes not associated with hallucination severity. Reduced between-module connectivity in the lateral occipital cortex, insula, and pars orbitalis and decreased within-module connectivity in the prefrontal, somatosensory, and primary visual cortices were associated with hallucination severity. Conversely, hallucination severity was associated with increased between- and within-module connectivity in the orbitofrontal and temporal cortex, as well as regions comprising the dorsal attentional and default mode network. These results suggest that hallucination severity is associated with marked alterations in structural network topology with changes in participation along the perceptual hierarchy. This may result in the inefficient transfer of information that gives rise to hallucinations in PD.

## Author Summary

Inefficient integration of information between external stimuli and internal perceptual predictions may lead to misperceptions or visual hallucinations in Parkinson’s disease (PD). In this study, we show that hallucinatory behavior in PD patients is associated with marked alterations in structural network topology. Severity of hallucinatory behavior was associated with decreased connectivity in a large subnetwork that included the majority of the diverse club, nodes with a high number of *between-module* connections. Furthermore, changes in *between-module* connectivity were found across brain regions involved in visual processing, top-down prediction centers, and endogenous attention, including the occipital, orbitofrontal, and posterior cingulate cortex. Together, these findings suggest that impaired integration across different sides across different perceptual processing regions may result in inefficient transfer of information.

## INTRODUCTION

Visual hallucinations (VHs) in Parkinson’s disease (PD) exist on a spectrum ranging from simple misperceptions to complex well-formed images (Barnes & David, 2001). With disease progression and loss of insight, VHs constitute a major source of distress for the patient (Goetz, 2009; Schrag, 2004) and comprise a high degree of burden for caregivers (Aarsland et al., 2007). Risk factors of VHs include older age and disease duration, sleep and mood disturbances, as well as cognitive decline (Barnes & David, 2001; Fénelon, Mahieux, Huon, & Ziégler, 2000; Lenka, Hegde, Arumugham, & Pal, 2017). Furthermore, previous work has shown that patients with VHs show disruptions in attentional processing (Hall et al., 2016), reduced performance on visuoperceptive tasks (Barnes, Boubert, Harris, Lee, & David, 2003; Gallagher et al., 2011; Ramírez-Ruiz, Junqué, Martí, Valldeoriola, & Tolosa, 2006), and decreased visual contrast sensitivity, color discrimination (Diederich et al., 1998), and acuity (Matsui et al., 2006). Current models of VHs have therefore focused on the interaction of perceptual and attentional dysfunction (for a review, see Muller, Shine, Halliday, & Lewis, 2014). Specifically, it has been proposed that failure to effectively integrate information from different processing sites across the perceptual hierarchy is likely to contribute to VHs and misperceptions in PD (Collerton, Perry, & McKeith, 2005; Diederich, Goetz, & Stebbins, 2005; Muller et al., 2014; Shine, Halliday, Carlos, Naismith, & Lewis, 2012).

Attention, prior experience, and expectations strongly influence perception. Perceptual predictions, generated from a myriad of modalities across the brain, guide perceptual processes to facilitate the interpretation of noisy and ambiguous input (Bar, 2009; Engel, Fries, & Singer, 2001; Summerfield et al., 2006). The orbitofrontal cortex (OFC) processes coarse information projected from the visual cortex and provides an “initial guess” of an object’s identity (Summerfield & Koechlin, 2008). Previous work in PD patients with VHs has shown that the accumulation of sensory evidence is slow and inefficient, which may result in an overreliance on these top-down predictions (O’Callaghan et al., 2017). Importantly, top-down visual processing regions can modulate neural activity in early visual regions, with expected stimuli leading to reduced activity (Meyer & Olson, 2011). Additionally, activity within the default mode network (DMN), a network involved in mediating endogenous perception, has shown to be increased during a misperception in this patient population (Shine, Halliday, et al., 2014). Therefore, VHs may arise when perceptual input is not properly integrated and internally generated images interfere with the perceptual process (Fletcher & Frith, 2008; Intaite, Noreika, Soliunas, & Falter, 2013; O’Callaghan et al., 2017; Powers, Kelley, & Corlett, 2016).

While functional neuroimaging studies have made significant contributions to our understanding (Hepp, Foncke, Olde Dubbelink, et al., 2017; Ramírez-Ruiz et al., 2008; Shine, Halliday, et al., 2014; Shine, Muller, et al., 2015; Yao et al., 2014), less is known about the involvement of white matter changes in the manifestation of VHs in PD. Experiments using diffusion tensor imaging (DTI) have reported altered white matter integrity in the optic nerve and optic radiation (Lee et al., 2016) as well as ascending tracts from the cholinergic nucleus basalis of Meynert to parietal and occipital cortical regions (Hepp, Foncke, Berendse, et al., 2017). However, given the involvement of large-scale brain networks in perception, unique insights into white matter changes associated with VHs can be gained by investigating whole-brain network topology. Topological features of the human connectome allow us to describe the arrangement of connections within and between segregated submodules (Bullmore & Sporns, 2009). Specifically, nodes that integrate these specialist communities are crucial for incorporating information streams of different modalities, which is essential for processes such as perception (Bertolero, Yeo, & D’Esposito, 2015; Muller, O’Callaghan, Walton, Shine, & Lewis, 2017). Therefore, investigating network topology can provide novel insights in changes across different perceptual hierarchies.

The current study aimed to examine whether VHs are associated with changes in structural network topology. To identify hallucinatory behavior in patients with PD, we assessed performance on a computerized task capable of inducing misperceptions (Shine et al., 2012), in combination with a validated questionnaire that evaluates hallucinatory behavior in PD patients (Shine, Mills, et al., 2015). We aimed to circumvent the sole reliance on self-reported symptom occurrence while controlling for the possibility of misclassifying hallucinators as nonhallucinators, when patients did not experience VHs during their clinic visit or when “passage” hallucinations go unreported. Additionally, by creating this composite score, we are able to assess visual hallucinatory severity, rather than classifying patients into artificial dichotomous patient groups.

We hypothesized that the severity of hallucinatory behavior would be associated with ineffective information processing as shown by reduced *between-module* scores in visual networks, reflecting reduced visual input to integration centers. Furthermore, increased *between-module* scores across top-down perceptual prediction areas and the DMN could indicate an overreliance on regions involved in the generation of internal percepts (Shine, O’Callaghan, Halliday, & Lewis, 2014).

## METHODS

Twenty-nine patients with idiopathic PD were included in this study. Demographic information including age, disease duration, and levodopa equivalence daily dose (LEDD) were obtained for all participants. All patients were assessed on the Hoehn & Yahr clinical stage (Hoehn & Yahr, 1998) and the motor aspect of the Movement Disorder Society Unified Parkinson’s Disease Rating Scale (MDS-UPDRS) part III (Goetz et al., 2008). Global cognition was assessed using the Mini-Mental State Examination (MMSE; Folstein, Robins, & Helzer, 1983), and set-shifting performance was assessed using the Trail Making Test Part B minus Part A (TMT_B-A_; Tombaugh, 2004). The study was approved by the ethics committee of the University of Sydney and was in accordance with the principles of the Helsinki Declaration. Written informed consent was obtained from all participants before participation.

### Bistable Percept Paradigm

**Figure 1.**
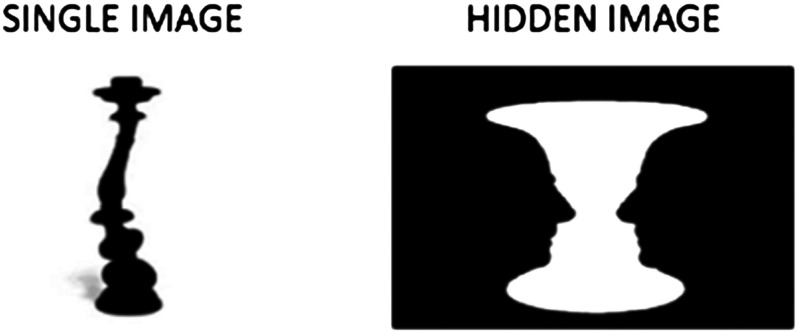
Example of single and hidden images of the BPP (Shine et al., 2012).

All patients performed the bistable percept paradigm (BPP; Shine et al., 2012), a behavioral task capable of inducing misperceptions in susceptible patients. In this task, patients were presented with either single or bistable percepts (i.e., “hidden” images as shown in Figure 1) for a maximum of 30 s in a randomized order. The participant had to decide whether the stimulus was a single or hidden image by a button press and describe to the examiner what they had seen. The recorded responses included the following: (a) correct single or correct hidden, (b) “missed,” recorded when the subject perceived a single image when a bistable percept was presented, and (c) “misperceptions,” recorded when a subject incorrectly identified a single image as a bistable image, that is, incorrectly reported an image that was not presented on the screen.

### Psychosis and Hallucinations Questionnaire

All patients completed the Psychosis and Hallucinations Questionnaire (PsycH-Q; Shine, Mills, et al., 2015). The PsycH-Q (Part A) consists of three subscales including (a) visual misperceptions, which includes questions about the presence of VHs, passage hallucinations, and three frequently reported contents of VHs including people, animals, and objects; (b) sensory misperceptions, including audition, touch, olfaction, and gustation; and (c) disordered thought and psychotic behavior. Participants rated the frequency of their symptoms on a 5-point Likert scale, ranging from 0 (“never experienced”) to 4 (“experienced daily”). The total score was calculated by summing the responses (Shine, Mills, et al., 2015; see Supporting Information, Hall et al., 2019). Part B of the PsycH-Q assesses symptoms related to VHs (i.e., attention and sleep) and was not included in this study.

### Composite Score

The percentage of misperceptions on the BPP (“indirect” measure of VH) and the total score on the PsycH-Q_A_ (“direct” measure of hallucinatory behavior) were standardized and then summed to create a composite score that reflected the severity of hallucinatory behavior (hereafter referred to as the hallucination severity score, HSS). The HSS was correlated with the demographic variables using parametric or nonparametric correlations depending on the distribution of the variables and was used as a correlate in the imaging analysis.

### MRI Acquisition

All participants underwent magnetic resonance imaging (MRI) using a 3-Tesla General Electric Discovery MR750 scanner (GE Medical Systems) with an 8-channel phased array head coil. Diffusion-weighted images (DWI) were obtained by using echo-planar imaging sequences with 61 different motion-probing gradient directions (TR/TE: 7,025/80 ms, 55 transverse slices, slice thickness: 2.5 mm, matrix: 256 × 256, FOV: 240 × 240 mm). The effective diffusion weighting was b = 1,000 s/mm^2^, and four volumes with no diffusion weighting (b = 0 s/mm^2^) were obtained at the beginning of each diffusion sequence. 3-D T1-weighted, anatomical images were obtained (TR/TE/TI: 7.2/2.7/450 ms, voxel size 1 × 1 × 1 mm, 196 transverse slices, 256 × 256 matrix, FOV: 256 × 256 mm, flip angle 12°). The 3-D T1 images were used for individual registration between T1-weighted anatomical and the DWI images and cortical parcellation using FreeSurfer (version 5.3; http://surfer.nmr.mgh.harvard.edu).

### Diffusion Tensor Imaging Preprocessing and Deterministic Fiber Tracking

DTI preprocessing was performed using the FMRIB Software Library (FSL, http://fsl.fmrib.ox.ac.uk). The preprocessing steps were as follows: (a) DTI images were corrected for susceptibility, head motion, and eddy current–induced geometrical distortions using FSL’s tool *eddy*; (b) a binary brain mask was created using *bet*; (c) images were realigned using a rigid body registration to the b = 0 image; then (d) a tensor was fitted in each voxel (Chang, Jones, & Pierpaoli, 2005), followed by (e) the computation of the fractional anisotropy (FA) level based on the eigenvalues for each voxel, in order to determine the preferred diffusion direction within a voxel. FA thus serves as a surrogate measure of white matter integrity, with lower levels of FA reflecting reduced white matter integrity (Sun et al., 2003; van den Heuvel & Sporns, 2011; Verstraete et al., 2010). The preferred diffusion direction information was then used to reconstruct the white matter tracts of the brain using a deterministic tracking approach based on the *fiber assignment by continuous tracking* (FACT) algorithm (Mori, Crain, Chacko, & van Zijl, 1999). Deterministic tractography yields less false positive tracts compared with probabilistic methods (Bastiani, Shah, Goebel, & Roebroeck, 2012). False positives are detrimental in network modularity as they occur more prevalently between than within modules (Zalesky et al., 2016). A streamline was started from eight seeds within each voxel of the brain (gray and white matter) following the main diffusion direction of the voxel and stopped when (a) the FA value < 0.1; (b) the traced fiber made a turn > 45°; or (c) the tract left the brain mask. The images were acquired when reverse phase-encoding direction approaches were not the standard procedure within acquisition protocols, which could have influenced the registration of diffusion and anatomical images. Therefore, anatomically constrained tractography was not applied (Smith, Tournier, Calamante, & Connelly, 2012). The atlas presented by Cammoun et al. (2012) was used, including 219 cortical regions and 14 subcortical regions. The weighted brain network was calculated for each participant, and consistency thresholding at 50% was applied (i.e., including the tracts found in 50% of the patients; de Reus & van den Heuvel, 2013). The mean density of the thresholded group matrix was 8.7%. To verify the results were not skewed by the choice of threshold, we also applied the thresholding method that retained most consistent edges across subjects but controlling for their distance (i.e., the consistency of edges within “bins” based on their length to avoid preferential retention of short edges; Misic et al., 2015). The mean density of the group matrix using this threshold was 13.2%.

### Network-Based Statistic

A network-based statistic (NBS) analysis was applied to investigate whether the HSS was associated with altered connectivity strength in an interconnected subnetwork of the brain (Zalesky, Fornito, & Bullmore, 2010). NBS is a nonparametric method for connectome-wide analysis, which aims to detect specific pairs of brain regions showing a significant effect of interest, while controlling for family-wise error (FWE) rate. Importantly, no inferences of individual connections are made; instead the null hypothesis can only be rejected at the subnetwork level. As such, NBS is similar to the cluster-based multiple-comparison approaches used in standard functional MRI analysis. To identify changes in subnetworks associated with the HSS, the *t* statistic was set at 1.7, determined using the critical value of the *t* distribution for our sample size (Field, 2009). Additionally, we verified the robustness of the results by controlling for disease severity using the MDS-UPDRS III (motor part) as covariate. Connections were deemed significant at FWE-corrected *p* value < 0.05 (one-sided) using 5,000 permutations.

To investigate whether the subnetwork involved particular functional networks, we investigated whether nodes in the subnetwork that correlated with the HSS overlapped with previously defined resting-state networks. To this end, seven canonical resting-state networks from the Yeo et al. (2011) atlas were overlaid with the structural parcellation and the percentage of nodes from each network included within the structural subnetwork that inversely related to HSS was calculated for each resting-state network. To analyze whether this overlap occurred significantly above chance, we randomly permuted the resting-state network identity of each region (5,000 iterations) and used the overlap between the randomized vector and the original node assignment to populate a null distribution. To test whether each individual resting-state network overlapped with the significant subnetwork, their overlap was compared with the null distributions. A resting-state network was identified as targeted if the true overlap was more than the 97.5th percentile of null distribution (i.e., the top 2.5%). A network was considered not to be associated with the HSS if the overlap was less than the 2.5th percentile of the null distribution.

### Graph Theoretical Analysis

The graph organizational measures were computed using the Brain Connectivity Toolbox (http://www.brain-connectivity-toolbox.net; Rubinov & Sporns, 2010). The thresholded, weighted brain networks were then partitioned into modules, which are nonoverlapping groups of highly connected nodes that are only sparsely connected with other modules, using the Louvain algorithm (Rubinov & Sporns, 2010). To account for the stochastic nature of the Louvain algorithm, a consensus partition was identified by calculating the module assignment for each node 500 times. To define an appropriate value for the resolution parameter (γ), the Louvain algorithm was iterated 100 times across a range of values (0.5–2.0 in steps of 0.1) of the group mean connectivity matrix and then the similarity of the resultant partitions was estimated using mutual information. The γ parameter of 1.9 provided the most robust estimates of topology across the iterations and was used to determine the optimal resolution of the network modularity.

After the nodes were assigned to their modules, their intra- and intermodular connectivity were calculated. Intramodular connectivity was calculated using the module degree z-score *W*_*i*_ (see Equation 1), in which a positive score reflects high *within-module* connections (compared with the node’s average number of connections), and negative z-scores denote the opposite. Intermodular connectivity was calculated using the participation coefficient *B*_*i*_ (see Equation 2). Low *B*_*i*_ values indicate few *between-module* connections, whereas high *B*_*i*_ values indicate uniformly distributed connections across modules (Hall, 2018). High *W*_*i*_ and high *B*_*i*_ scores are not mutually exclusive (Guimerà & Nunes Amaral, 2005).

$$W_{i}=\frac{\kappa_{i}-\kappa_{s_{i}}}{\sigma_{\kappa_{{\text{s}}_{\text{i}}}}}$$ (1)

**Equation 1:** Module degree z-score W_*i*_, where κ_*i*_ is the strength of the connections of region *i* to other regions in its module s_i_, κ_s_i__ is the average of κ over all the regions in s_*i*_, and σ_κ_*s*_*i*___ is the standard deviation of κ in s_i_.

$$B_{i}=1-\sum_{s=1}^{n_{M}}{\left(\frac{\kappa_{is}}{\kappa_{i}}\right)}^{2}$$ (2)

**Equation 2:** Participation coefficient *B*_*i*_, where κ_is_ is the strength of the positive connections of region *i*to regions in module *s*, and κ_i_ is the sum of strengths of all positive connections of region *i*. The participation coefficient of a region is therefore close to 1 if its connections are uniformly distributed among all the modules and 0 if all of its links are within its own module.

To test whether nodes within the subnetwork identified using the NBS analysis differed from nodes not included in the subnetwork, the average *W*_*i*_ and *B*_*i*_ of the subnetwork were contrasted against the average *W*_*i*_ and *B*_*i*_ of the nodes not included in the subnetwork using nonparametric permutation testing.

To test whether the HSS correlated with the *W*_*i*_ and *B*_*i*_ nodes across the whole-brain connectome, a Spearman’s rho correlation was performed followed by a nonlinear permutation test using 5,000 iterations to control for multiple comparisons (Nichols & Holmes, 2002), using an alpha of 0.05. This approach was repeated using the different threshold (Misic et al., 2015), and the outcome was correlated to the *W*_*i*_ and *B*_*i*_ using the original threshold. Both the *W*_*i*_ and the *B*_*i*_ scores calculated using the aforementioned threshold highly correlated with the *W*_*i*_ and *B*_*i*_ scores calculated with the consensus threshold (*r* = 0.92 and *r* = 0.94, respectively), indicating that the results were not biased by the chosen thresholding method.

### Diverse Club Analysis

We identified the “diverse club” of the network, which comprised the top 20% of *B*_*i*_ nodes (Bertolero, Yeo, & D’Esposito, 2017). These nodes play an important role in network integration, and changes to these nodes could affect between-module communication (Bertolero et al., 2017). We normalized the diverse club coefficient in reference to a null model: A random vector with a preserved modular structure was created by randomizing the mean participation coefficient of each node for 5,000 iterations. The diverse club was identified as those regions with a participation coefficient greater than the 95th percentile of the permuted distribution. We investigated whether the number of diverse club nodes was significantly higher within the subnetwork associated with the HSS, compared with nodes that were not included in the subnetwork identified using the NBS analysis.

## RESULTS

### Demographics

Table 1 presents the descriptive variables of the 29 patients. The mean percentage of misperceptions on the BPP was 18.48 (range: 0–49), and the mean score on the PsycH-Q_*A*_ was 9.48 (range: 0–34, max score = 52), highlighting a diverse range of hallucinatory behavior in the patient cohort. The PsycH-Q_*A*_ and the BPP score showed a positive, significant correlation (*r* = 0.52, *p* = 0.004). Finally, to verify the severity score to the “gold standard,” we correlated the HSS in a large cohort of patients with PD and Lewy body dementia (*n* = 75) to the MDS-UPDRS item 2 and found a correlation of *r* = 0.53 (*p* < 0.001). However, given higher construct validity (Shine, Mills, et al., 2015), we opted to include the PsycH-Q_A_ and BP scores in the composite score (HSS) for the remainder of our analysis.

**Table 1.** Demographics and clinical variables

**Variable**	**Mean (range)**	**Correlation with HSS *r* (*p* value)**
Age (y)	66.8 ± 8 (51–84)	0.18 (0.345)
Duration (y)	5.8 ± 4 (1.2–16)	−0.05 (0.785)
LEDD	617.8 ± 392 (125–1548)	−0.06 (0.767)
H & Y	1.9 ± 0.5 (1–3)	0.01 (0.949)
MDS-UPDRS III	30.0 ± 14 (7–55)	0.29 (0.125)
MMSE	28.5 ± 2 (25–30)	−0.06 (0.762)
TMT_B-A_	78.9 ± 65 (−1–123)	0.33 (0.080)
BPP % misperceptions	18.5 ± 17 (0–49)	–
PsycH-Q_A_	9.5 ± 8 (0–34)	–

LEDD = levodopa equivalence daily dose; MDS-UPDRS III = motor part of the Movement Disorder Society Unified Parkinson’s Disease Rating Scale; H & Y = Hoehn and Yahr; MMSE = Mini-Mental State Examination; TMT_B-A_ = Trail Making Test Part B – Part A; BPP = bistable percept paradigm; PsycH-Q_A_ = Psychosis and Hallucinations Questionnaire, Part A.

The HSS showed a positive correlation trending towards significance with the TMT_B-A_ (*r* = 0.33, *p* = 0.08). No significant correlations were observed between the HSS and other demographic and clinical variables.

### The HSS Correlated With Decreased Connectivity in a Large Subnetwork

**Figure 2.**
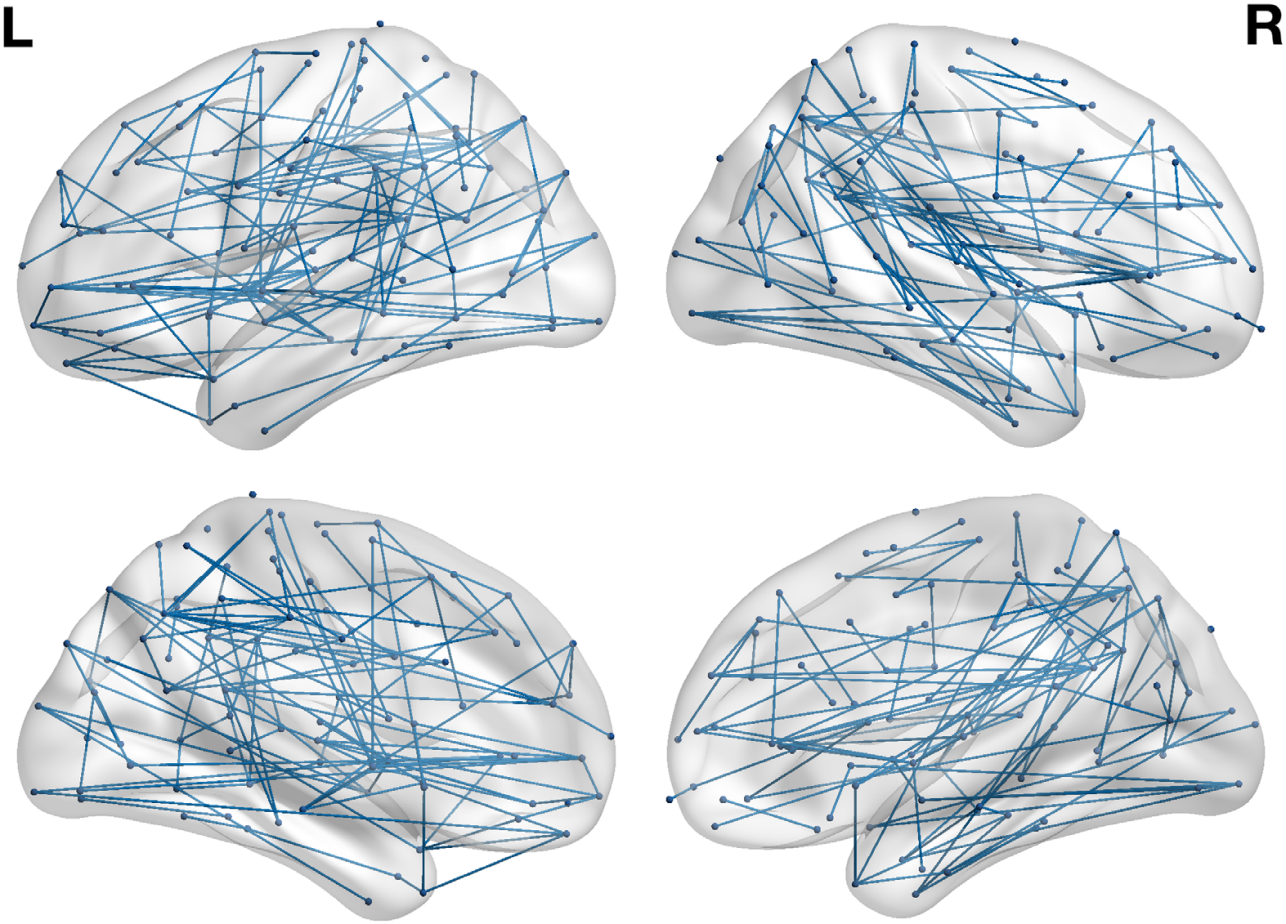
NBS analysis reveals a subnetwork, comprising 183 edges and 127 nodes with reduced connectivity strength correlated to increased HSS (*p* < 0.05). Figure visualized with BrainNet Viewer (Xia, Wang, & He, 2013).

As illustrated in Figure 2, the NBS analysis revealed a subnetwork comprising 183 edges (8% of the edges in the thresholded connectivity matrix) and 127 nodes with reduced FA-based connectivity strength correlated to the HSS (*p* < 0.05). Using disease severity as a covariate, the NBS analysis revealed a similar subnetwork comprising 177 edges and 135 nodes that showed a correlation with the HSS that was trending towards significance (*p* = 0.059). The effects presented with a fairly liberal threshold, suggesting the changes related to the HSS are subtle yet topological extended (Zalesky et al., 2010). The size of networks identified using a range of *t* statistics are presented in the Supporting Information (Hall et al., 2019). No significant subnetwork was identified in the opposite direction (positive correlation between the HSS and connectivity strength). Furthermore, the group average *B*_*i*_ score within the subnetwork was 0.506, which was significantly higher (*p* < 0.05) than nodes outside this network, which show a group average of 0.310. The group average *W*_*i*_ score of the nodes within the network was higher than the group average *W*_*i*_ score of nodes outside the network (0.149 and −0.178, respectively), yet this difference did not remain significant when controlling for disease severity (*p* = 0.104).

### The Subnetwork Includes All Subcortical Nodes but Did Not Target a Specific Cortical Resting-State Network

**Figure 3.**
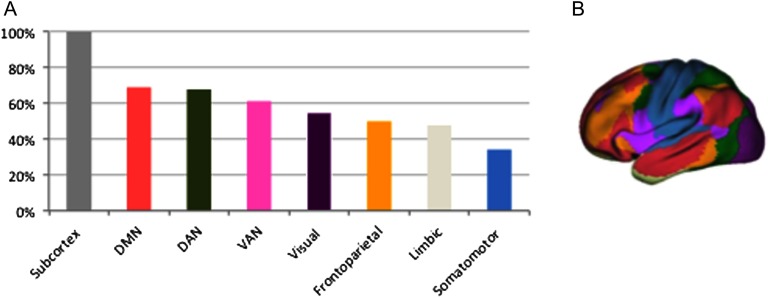
Overlap between the identified structural subnetwork and functional resting-state networks. (A) The percentage of nodes included in the subnetwork for each resting-state network. DMN = default mode network; VAN = ventral attentional network; DAN = dorsal attentional network. (B) The functional resting-state networks of the Yeo et al. (2011) atlas.

The subnetwork that showed decreased connectivity strength correlated with the HSS included all 14 subcortical nodes (*p* < 0.05). As illustrated in Figure 3, the subnetwork further included nodes across the cortex. However, none of the other the resting-state networks were significantly correlated with the HSS (*p* > 0.05), yet the somatomotor network was relatively spared (*p* < 0.05).

### Nodes in the Subnetwork Show High Participation Scores

**Figure 4.**
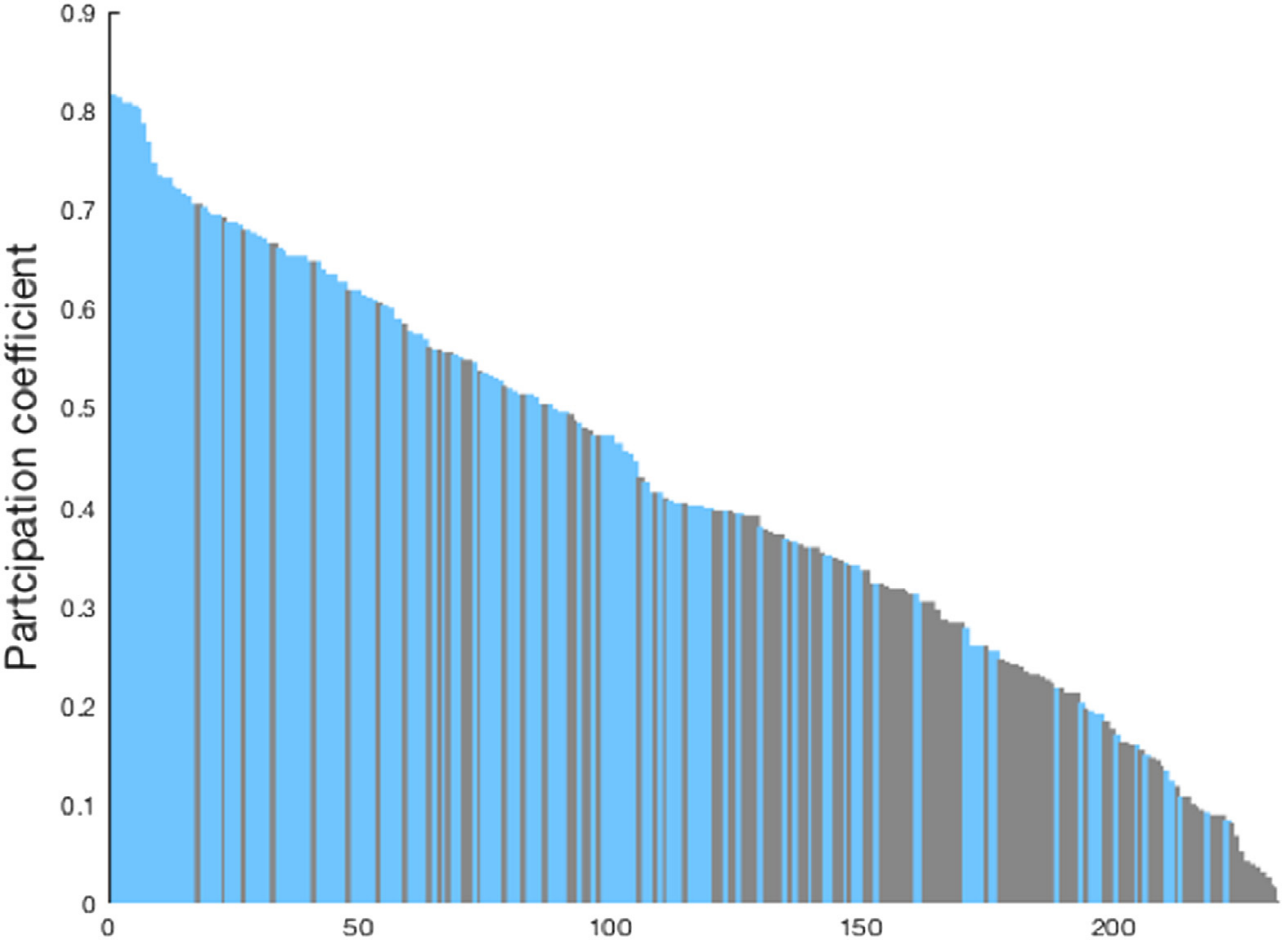
Nodes ranked according to the *B*_*i*_ scores. Blue: nodes included in the subnetwork; gray: nodes not included in the subnetwork correlated to the HSS.

Eighteen nodes were included in the diverse club (see Supporting Information, Hall et al., 2019). Seventeen of the eighteen nodes (94%) of the diverse club were included in the aforementioned subnetwork, which was deemed significantly above chance (*p* < 0.001). As illustrated in Figure 4, nodes with high participation coefficients were more often part of the subnetwork.

### The HSS Correlated With *W*_*i*_ and *B*_*i*_ Scores

**Figure 5.**
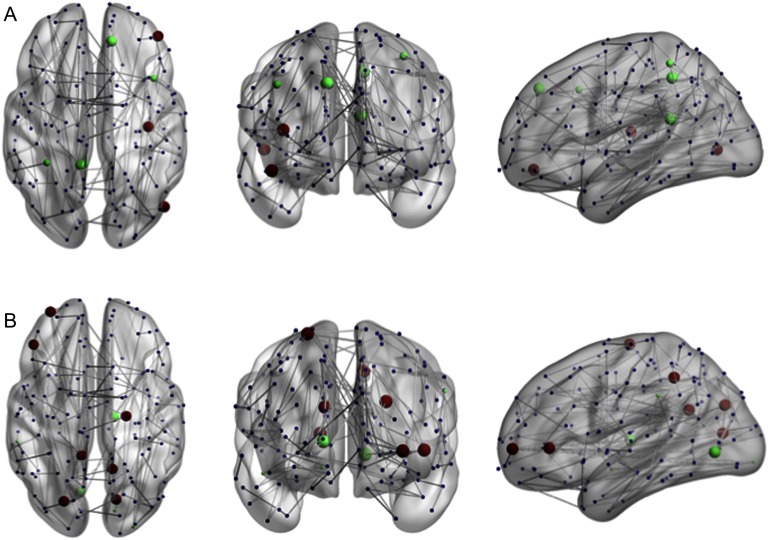
Significant changes in *between*- and *within*-module scores associated with the HSS. (A) Nodes with a significant correlation between participation coefficients and the HSS. (B) Nodes with a significant correlation between the module degree z-score and HSS. Green indicates a positive correlation, red indicates a negative correlation. Larger nodes are part of the subnetwork. Figure visualized with BrainNet Viewer (Xia et al., 2013).

When investigating the whole structural connectome, the HSS positively correlated to regional *B*_*i*_ (i.e., higher participation scores were associated with higher severity values) for nodes in the left medial OFC, a node in the right anterior and left posterior cingulate, precuneus, and the caudal middle frontal gyrus. Furthermore, nodes in the right occipital, pars orbitalis, and insula showed negative correlations between the HSS and participation coefficient (i.e., lower participation scores were associated with higher scores on the HSS; see Table 2 and Figure 5). However, when controlling for disease severity, the insula and medial OFC were only trending towards significance, while the lateral occipital cortex and anterior posterior cingulate did not remain significant.

**Table 2.** Spearman’s rho correlation between the participation coefficient and the HSS (*p* < 0.05; permutation test)

**Node**	**X**	**Y**	**Z**	**Rho**	Rho*	**Subnetwork**
**Positively correlated**						
*Frontal*						
ctx-lh-medialorbitofrontal_2	−5	33	−20	0.37	0.35^#^	✓
ctx-rh-caudalmiddlefrontal_2	40	15	39	0.43	0.53	–
*Parietal*						
ctx-lh-precuneus_2	−10	−44	46	0.40	0.37	✓
*Cingulate*						
ctx-lh-posteriorcingulate_2	−8	−43	21	0.57	0.53	✓
ctx-rh-superiorfrontal_3	11	40	40	0.36	0.31ˆ	✓
**Negatively correlated**						
*Frontal*						
ctx-rh-parsorbitalis_1	43	43	−10	−0.46	−0.46	✓
*Occipital*						
ctx-rh-lateraloccipital_5	47	−73	2	−0.39	−0.21ˆ	✓
*Insular*						
ctx-rh-insula_1	36	−18	13	−0.40	−0.32^#^	✓

* Rho after controlling for disease severity;

# *p* < 0.1;

ˆ *p* > 0.1.

ctx-lh = left hemisphere; ctx-rh = right hemisphere.

Increased HSS scores were further associated with increased module degree z-scores in the right thalamus, bilateral lingual, left medial OFC, pars opercularis, supramarginal gyrus, and superior temporal cortices. The right lateral occipital cortex also showed a positive correlation, but this did not remain significant after controlling for disease severity. Decreased HSS scores were associated with increased module degree z-scores in the bilateral precuneus, left parts triangularis, rostral middle frontal, and superior parietal cortex. The right pericalcarine and precentral gyrus also a showed negative correlation with the HSS, yet this did not remain significant after controlling for disease severity (see Table 3 and Figure 5).

**Table 3.** Spearman’s rho correlation between the module degree z-score and the HSS (*p* < 0.05; permutation test)

**Node**	**X**	**Y**	**Z**	**Rho**	Rho*	**Subnetwork**
**Positively correlated**						
*Subcortical*						
Right-Thalamus-Proper	13	−17	7	0.49	0.45	✓
*Frontal*						
ctx-lh-medialorbitofrontal_1	−7	52	−11	0.40	0.43	–
ctx-lh-parsopercularis_2	−47	14	15	0.41	0.44	–
*Parietal*						
ctx-lh-supramarginal_3	−55	−34	35	0.45	0.53	–
*Temporal*						
ctx-rh-superiortemporal_4	46	−6	−11	0.57	0.47	–
*Occipital*						
ctx-rh-lateraloccipital_3	27	−94	−6	0.39	0.34^#^	–
ctx-lh-lingual_2	−10	−70	0	0.42	0.50	✓
ctx-rh-lingual_1	14	−83	−7	0.41	0.37	–
**Negatively correlated**						
*Frontal*						
ctx-lh-parstriangularis_1	−43	33	2	−0.50	−0.43	✓
ctx-lh-rostralmiddlefrontal_5	−31	56	2	−0.57	−0.50	✓
ctx-rh-precentral_6	22	−17	67	−0.39	−0.35^#^	✓
*Parietal*						
ctx-lh-precuneus_2	−10	−44	46	−0.46	−0.43	✓
ctx-lh-superiorparietal_6	−22	−76	29	−0.41	−0.47	✓
ctx-rh-precuneus_2	12	−54	26	−0.39	−0.43	✓
*Occipital*						
ctx-rh-pericalcarine_1	15	−75	12	−0.38	−0.24ˆ	✓

* Rho after controlling for disease severity;

# *p* < 0.1;

ˆ *p* > 0.1.

ctx-lh = left hemisphere; ctx-rh = right hemisphere.

## DISCUSSION

The aim of this study was to determine whether changes in structural network topology were associated with hallucinatory behavior in PD. We showed that severity of hallucinatory behavior was negatively correlated with connectivity across a bilateral subnetwork. Regions within this subnetwork showed higher participation compared with regions outside this network. The loss of connectivity strength may force the system to adapt and reroute information across less efficient pathways, impeding the standard sensory integration process. Importantly, 94% of the nodes in the diverse club were included in this subnetwork. This community of high participation nodes is thought to control the integration of relatively segregated regions (Bertolero et al., 2017). Indeed, the diverse connectivity pattern of these nodes makes them crucial for the functional coordination of brain regions during tasks, and activity in these nodes predicts changes in the coupling of other regions (Bertolero et al., 2017). Severity of hallucinatory behavior may thus be the result of impaired integration and segregation of brain networks or “modules,” affecting effective information transfer. Finally, we showed regional changes in participation associated with hallucination severity (the HSS score), with a positive correlation between participation scores in the medial OFC, cingulate, precuneus, and middle frontal gyrus and the HSS and negative correlation with participation scores in the lateral occipital cortex, pars orbitalis, and insula. These findings suggest a reweighting of the regions along the perceptual hierarchy, which may give rise to hallucinations.

Lower participation of the lateral occipital cortex may reflect reduced early visual processing, resulting in ineffective accumulation of visual information from the environment. Previous work using a Bayesian drift diffusion model has demonstrated that accumulation speed and quality of perceptual information are reduced in PD patients with VH (O’Callaghan et al., 2017). Furthermore, reduced quality or integration of visual stimuli may increase perceptual uncertainty, a suggestion that aligns with increased participation in the dorsal anterior cingulate cortex (Keri, Decety, Roland, & Gulyas, 2004). Perceptual uncertainty may place excessive emphasis on top-down prediction centers, which subsequently could lead to a reduced activity in early visual regions (Meyer & Olson, 2011). However, the reduced participation score in the occipital and anterior cingulate cortex may not be specific to hallucinatory behavior, as the negative correlations did not remain significant after controlling for disease severity.

This emphasis on top-down visual processing centers is supported by the increased participation coefficient and module degree z-score in the medial OFC. The OFC has an integrative function across brain networks, as evidenced by its high participation coefficient. Additionally, this region is thought to facilitate recognition during visual perception by integrating incoming sensory information with previous experiences and expectations (Panichello, Cheung, & Bar, 2012). During typical visual perception, the OFC is activated early in response to visual stimuli, receiving low spatial frequency signals from the visual cortex (Bar et al., 2006). Notably, only stimuli resembling known objects are shown to activate the OFC, which in turn generates a semantic association and provides a predictive signal to the visual system (Chaumon, Kveraga, Barrett, & Bar, 2014). Conversely, visual stimuli that carry no meaningful association do not activate the OFC in healthy individuals. Hence, it could be speculated that because of decreased quality of visual input, inappropriate recruitment of the OFC occurs, which may result in ascribing false associative information to visual stimuli.

The manifestation of VHs has previously been recognized as a dysfunction between the attentional networks (Shine, Muller, et al., 2015). Specifically, patients with VHs are shown to be less able to recruit the dorsal attentional network (DAN), which enables the selection of appropriate sensory stimuli (Corbetta, Patel, & Shulman, 2008). With reduced control of this network, ambiguous stimuli might instead be interpreted by the ventral attentional network (VAN) and the DMN, which are less well equipped for this task. Our results showed increased participation in the posterior cingulate cortex (PCC), a key hub of the DMN. PCC activity has been implicated in regulating the focus of attention, specifically the shift from the external world into internal mentation (Weissman, Roberts, Visscher, & Woldorff, 2006). Furthermore, the PCC is involved in mind wandering and supports internally directed cognition (Leech & Sharp, 2013). A failure to suppress PCC activity may lead to the intrusion of internal thoughts into task performance (Sonuga-Barke & Castellanos, 2007). Moreover, a positive correlation was found between the HSS and the module degree z-score of the left pars opercularis, a node in the VAN, a network that is activated when expectations in perception are violated (Corbetta & Shulman, 2002; Shine, Halliday, et al., 2014). Conversely, a negative correlation between the HSS scores and module degree score and participation coefficients was found in other nodes of the VAN, namely the left pars triangularis, the right pars orbitalis, and insula. The left pars triangularis supports resolving competition between simultaneously active representations (Badre & Wagner, 2007), while the insula plays an important role in dynamically shifting attention between the attentional control networks (Menon & Uddin, 2010). The anterior insula has previously been shown to be reduced in volume in PD patients with VH (Ibarretxe-Bilbao et al., 2010; Shine, Halliday, et al., 2014). Together, these results suggest that ineffective communication between attentional networks in the brain may predispose an individual to hallucinate. Surprisingly, the participation coefficient of a node within the DAN (“ctx-rh-caudalmiddlefrontal_2”) showed a positive correlation with the HSS. This node was not part of the subnetwork, and it may be possible that this is a compensatory response to the loss of connectivity strength in the other DAN regions. Notably, the connectivity matrix shows between module connections of this region with nodes in the somatomotor and the frontoparietal network, but not with the DMN or VAN.

Finally, all nodes that showed negative correlations with the HSS were included in the subnetwork. Decreased *within-module* scores were found across the prefrontal and the somatosensory association cortex, as well as in the primary visual cortex, while there was a positive correlation between the HSS and the bilateral secondary visual cortex, perhaps as a result of the decreased visual input from V1. Additionally, the supramarginal gyrus, a node that has been shown to be functionally active during spatial perception but also during visual imagery (Ganis, Thompson, & Kosslyn, 2004), showed an increased module degree z-score with increasing severity of VHs. Furthermore, a positive correlation with the HSS and the module degree score in the superior temporal cortex, a region involved in auditory processing, was found. It could be speculated that increased visual uncertainty may stimulate other sensory processing areas. Conversely, previous work in schizophrenia has shown increased activation in the superior temporal cortex during a mismatch between expected and perceived auditory feedback (Fu et al., 2008). The HSS also included hallucinations of other sensory modalities, and it could be speculated that a greater emphasis is being placed on predictions of auditory stimuli, as reflected as increased *within-module* connectivity in the superior temporal cortex.

This study has several limitations worth noting. First, the DWI data were acquired without EPI distortion correction. This may have affected the accuracy of registration between DWI and T1 images in the frontal and temporal cortices. Because of relatively low diffusion weighting used in the current MRI protocol, it was chosen to employ DTI rather than more sophisticated methods such as constrained spherical deconvolution, an algorithm that more adequately deals with multiple fiber directions within one voxel than DTI. Furthermore, after controlling for disease severity, the identified subnetwork was only trending towards significance. The correlation between the HSS and the participation coefficients of the lateral occipital and anterior cingulate did not remain significant, while the OFC and insula were only trending towards significance, and similar patterns were found for the module degree z-score: correlation with the HSS and nodes in the occipital and precentral regions did not remain significant. However, this may be expected given the close relationship between overall symptom severity and phenotypic symptom severity. Importantly, the correlation coefficients showed notable consistency across the analyses, suggesting the HSS was able to distinguish interindividual variability. While the current study included PD patients with no self-reported hallucinations (*n* = 6), no data from a healthy control group were acquired, which could have aided in disassociating the significant network measures and disease burden. The LEDD score was not associated with the HSS in the current study, and the relation between hallucinations and a LEDD-related side effect has been dismissed in previous work (Fénelon et al., 2000; Graham, Grünewald, & Sagar, 1997; Sanchez-Ramos, Ortollm, & Paulson, 1996; Shergill, Walker, & Le Katona, 1998). Furthermore, hallucinations may occur in the absence of dopaminergic treatment in PD or dementia with Lewy bodies (Ala, Yang, Sung, & Frey, 1997). Therefore, the current study chose not to include LEDD score as a covariate. Furthermore, the investigation was conducted in a relatively small group of PD patients, and future studies should replicate our findings in a larger sample size. Finally, this study chose to include the PsycH-Q to assess hallucinations. This is an in-depth questionnaire with high construct validity; however, patients have to report their hallucinations themselves. Ideally, a trained neurologist or qualified researcher, who can probe the patients when in doubt, should assist with this process.

## CONCLUSIONS

We cautiously conclude that hallucinatory behavior in PD patients is associated with marked alterations in structural network topology. Severity of hallucinatory behavior was associated with decreased connectivity in a large subnetwork that included the majority of the diverse club. These changes may result in an inefficient rerouting of information across less efficient pathways, which may lead to impaired visual integration processes. Furthermore, nodes within the orbitofrontal cortex and temporal lobes showed increased participation scores, while the visual association cortex, insula, and middle frontal gyrus showed decreased scores associated with the HSS score. These findings suggest that impaired integration across different regions along the perceptual hierarchy may result in inefficient transfer of information. A failure to effectively switch between attentional networks and the intrusion of internal percepts could give rise to perceptual glitches, such as misperceptions and hallucinations.

## ACKNOWLEDGMENTS

We thank the patients and their families who contribute to our research at the Parkinson’s Disease Research Clinic. We thank Dr. Váša for sharing his thresholding code (https://github.com/frantisekvasa/matlab_general). The DWI data were processed during the 10kin1day initiative at the Dutch Connectome Lab. This research was supported by Sydney Informatics Hub, funded by the University of Sydney.

## AUTHOR CONTRIBUTIONS

Julie M. Hall: Conceptualization; Data curation; Formal analysis; Investigation; Methodology; Project administration; Visualization; Writing – original draft. Claire O’Callaghan: Data curation; Investigation; Methodology; Writing – review & editing. Alana Justine Muller: Data curation; Writing – review & editing. Kaylena A. Ehgoetz Martens: Writing – review & editing. Joseph R. Phillips: Writing – review & editing. Ahmed A. Moustafa: Writing – review & editing. Simon J. G. Lewis: Funding acquisition; Investigation; Supervision; Writing – review & editing. James M. Shine: Conceptualization; Data curation; Formal analysis; Investigation; Methodology; Supervision; Writing – review & editing.

## FUNDING INFORMATION

Claire O’Callaghan, National Health and Medical Research Council Neil Hamilton Fairley Fellowship, Australia, Award ID: 1091310. Simon J. G. Lewis, National Health and Medical Research Council of Australia, Award ID: 1037746. Simon J. G. Lewis, National Health and Medical Research Council of Australia, Award ID: 1095127. Simon J. G. Lewis, National Health and Medical Research Council of Australia and the Australian Research Council, Dementia Fellowship, AWARD ID: 1110414. James M. Shine, National Health and Medical Research Council Project Grant, Australia, Award ID: 1156536. James M. Shine, The University of Sydney Robinson Fellowship.

## TECHNICAL TERMS

MDS Unified Parkinson’s Disease Rating Scale: A commonly used measure of Parkinson’s disease clinical symptom severity.
Bistable percept paradigm: A computer-based task using bistable percepts to measure visual misperceptions.
Psychosis and Hallucinations Questionnaire: A 20-item self-reported questionnaire to measure hallucinatory phenomenology.
Tractography: Three-dimensional representation of the brain’s white matter tracts derived from diffusion MRI data.
Network-based statistic: A statistical method to identify connections and networks comprising the connectome associated with behavior.
Module degree z-score: A measure of diversity of intramodular connections of individual nodes.
Participation coefficient: A measure of diversity of intermodular connections of individual nodes.
Diverse club: A subset of nodes in a network with high participation coefficients that exhibit properties consistent with an integrative network function.
Structural connectome: Brain connectivity graphs obtained from diffusion MRI and tractography.

## References

[bib1] Aarsland D., Bronnick K., Ehrt U., De Deyn P. P., Tekin S., Emre M., & Cummings J. L. (2007). Neuropsychiatric symptoms in patients with Parkinson’s disease and dementia: Frequency, profile and associated care giver stress. Journal of Neurology, Neurosurgery, and Psychiatry, 78(1), 36–42. 10.1136/jnnp.2005.083113 PMC2117797 16820421

[bib2] Ala T. A., Yang K. H., Sung J. H., & Frey W. H. (1997). Hallucinations and signs of parkinsonism help distinguish patients with dementia and cortical Lewy bodies from patients with Alzheimer’s disease at presentation: A clinicopathological study. Journal of Neurology, Neurosurgery, and Psychiatry, 62(1), 16–21. 10.1136/jnnp.62.1.16 PMC486689 9010394

[bib3] Badre D., & Wagner A. D. (2007). Left ventrolateral prefrontal cortex and the cognitive control of memory. Neuropsychologia, 45(13), 2883–2901. 17675110 10.1016/j.neuropsychologia.2007.06.015

[bib4] Bar M. (2009). The proactive braerin: Memory for predictions. Philosophical Transactions of the Royal Society B: Biological Sciences, 364(1521), 1235–1243. 10.1098/rstb.2008.0310 PMC2666710 19528004

[bib5] Bar M., Kassam K. S., Ghuman A. S., Boshyan J., Schmid A. M., Dale A. M., … Halgren E. (2006). Top-down facilitation of visual recognition. Proceedings of the National Academy of Sciences, 103(2), 449–454. 10.1073/pnas.0507062103 PMC1326160 16407167

[bib6] Barnes J., Boubert L., Harris J., Lee A., & David A. S. (2003). Reality monitoring and visual hallucinations in Parkinson’s disease. Neuropsychologia, 41(5), 565–574. 12559149 10.1016/s0028-3932(02)00182-3

[bib7] Barnes J., & David A. (2001). Visual hallucinations in Parkinson’s disease: A review and phenomenological survey. Journal of Neurology, Neurosurgery, and Psychiatry, 70(6), 727–733. 10.1136/jnnp.70.6.727 PMC1737396 11385004

[bib8] Bastiani M., Shah N. J., Goebel R., & Roebroeck A. (2012). Human cortical connectome reconstruction from diffusion weighted MRI: The effect of tractography algorithm. NeuroImage, 62(3), 1732–1749. 22699045 10.1016/j.neuroimage.2012.06.002

[bib9] Bertolero M. A., Yeo B. T. T., & D’Esposito M. (2015). The modular and integrative functional architecture of the human brain. Proceedings of the National Academy of Sciences, 112(49), E6798–E6807. 10.1073/pnas.1510619112 PMC4679040 26598686

[bib10] Bertolero M. A., Yeo B. T. T., & D’Esposito M. (2017). The diverse club. Nat Commun, 8(1), 1277 29097714 10.1038/s41467-017-01189-w PMC5668346

[bib11] Bullmore E., & Sporns O. (2009). Complex brain networks: Graph theoretical analysis of structural and functional systems. Nature Reviews Neuroscience, 10(3), 186. 19190637 10.1038/nrn2575

[bib12] Cammoun L., Gigandet X., Meskaldji D., Thiran J. P., Sporns O., Do K. Q., … Hagmann P. (2012). Mapping the human connectome at multiple scales with diffusion spectrum MRI. Journal of Neuroscience Methods, 203(2), 386–397. 22001222 10.1016/j.jneumeth.2011.09.031

[bib13] Chang L. C., Jones D. K., & Pierpaoli C. (2005). RESTORE: Robust estimation of tensors by outlier rejection. Magnetic Resonance in Medicine, 53(5), 1088–1095. 15844157 10.1002/mrm.20426

[bib14] Chaumon M., Kveraga K., Barrett L. F., & Bar M. (2014). Visual predictions in the orbitofrontal cortex rely on associative content. Cerebral Cortex, 24(11), 2899–2907. 23771980 10.1093/cercor/bht146 PMC4193460

[bib15] Collerton D., Perry E., & McKeith I. (2005). Why people see things that are not there: A novel Perception and Attention Deficit model for recurrent complex visual hallucinations. Behavioral and Brain Sciences, 28(6), 737–757; discussion 757–794 16372931 10.1017/S0140525X05000130

[bib16] Corbetta M., Patel G., & Shulman G. L. (2008). The reorienting system of the human brain: From environvment to theory of mind. Neuron, 58(3), 306–324. 18466742 10.1016/j.neuron.2008.04.017 PMC2441869

[bib17] Corbetta M., & Shulman G. L. (2002). Control of goal-directed and stimulus-driven attention in the brain. Nature Reviews Neuroscience, 3(3), 201–215. 11994752 10.1038/nrn755

[bib18] de Reus M. A., & van den Heuvel M. P. (2013). Estimating false positives and negatives in brain networks. NeuroImage, 70, 402–409. 23296185 10.1016/j.neuroimage.2012.12.066

[bib19] Diederich N. J., Goetz C. G., Raman R., Pappert E. J., Leurgans S., & Piery V. (1998). Poor visual discrimination and visual hallucinations in Parkinson’s disease. Clinical Neuropharmacology, 21(5), 289–295. 9789709

[bib20] Diederich N. J., Goetz C. G., & Stebbins G. T. (2005). Repeated visual hallucinations in Parkinson’s disease as disturbed external/internal perceptions: Focused review and a new integrative model. Movement Disorders, 20(2), 130–140. 15486924 10.1002/mds.20308

[bib21] Engel A. K., Fries P., & Singer W. (2001). Dynamic predictions: Oscillations and synchrony in top-down processing. Nature Reviews Neuroscience, 2, 704 11584308 10.1038/35094565

[bib22] Fénelon G., Mahieux F., Huon R., & Ziégler M. (2000). Hallucinations in Parkinson’s disease: Prevalence, phenomenology and risk factors. Brain, 123(4), 733–745. 10734005 10.1093/brain/123.4.733

[bib23] Field A. (2009). Discovering statistics using SPSS. London, UK: SAGE Publications.

[bib24] Fletcher P. C., & Frith C. D. (2008). Perceiving is believing: A Bayesian approach to explaining the positive symptoms of schizophrenia. Nature Reviews Neuroscience, 10, 48 19050712 10.1038/nrn2536

[bib25] Folstein M. F., Robins L. N., & Helzer J. E. (1983). The mini-mental state examination. Archives of General Psychiatry, 40(7), 812. 6860082 10.1001/archpsyc.1983.01790060110016

[bib26] Fu C. H. Y., Brammer M. J., Yágüez L., Allen P., Matsumoto K., Johns L., … van Haren N. (2008). Increased superior temporal activation associated with external misattributions of self-generated speech in schizophrenia. Schizophrenia Research, 100(1), 361–363. 18042348 10.1016/j.schres.2007.10.023

[bib27] Gallagher D. A., Parkkinen L., O’Sullivan S. S., Spratt A., Shah A., Davey C. C., … Lees A. J. (2011). Testing an aetiological model of visual hallucinations in Parkinson’s disease. Brain, 134(11), 3299–3309. 21921019 10.1093/brain/awr225

[bib28] Ganis G., Thompson W. L., & Kosslyn S. M. (2004). Brain areas underlying visual mental imagery and visual perception: An fMRI study. Cognitive Brain Research, 20(2), 226–241. 15183394 10.1016/j.cogbrainres.2004.02.012

[bib29] Goetz C. G. (2009). Scales to evaluate psychosis in Parkinson’s disease. Parkinsonism and Related Disorders, 15(Suppl. 3), S38–S41. 10.1016/S1353-8020(09)70777-1 20083004

[bib30] Goetz C. G., Tilley B. C., Shaftman S. R., Stebbins G. T., Fahn S., Martinez-Martin P., … Dodel R. (2008). Movement Disorder Society–sponsored revision of the Unified Parkinson’s Disease Rating Scale (MDS-UPDRS): Scale presentation and clinimetric testing results. Movement Disorders, 23(15), 2129–2170. 19025984 10.1002/mds.22340

[bib31] Graham J. M., Grünewald R. A., & Sagar H. J. (1997). Hallucinosis in idiopathic Parkinson’s disease. Journal of Neurology, Neurosurgery, and Psychiatry, 63(4), 434–440. 10.1136/jnnp.63.4.434 PMC2169767 9343119

[bib32] Guimerà R., & Nunes Amaral L. A. (2005). Functional cartography of complex metabolic networks. Nature, 433, 895 15729348 10.1038/nature03288 PMC2175124

[bib33] Hall J. M. (2018). Topology: Structural network topology code for “Alterations in structural network topology contribute to freezing of gait in Parkinson’s disease,” GitHub. https://github.com/juliemaehall/topology

[bib34] Hall J. M., O’Callaghan C., Muller A. J., Ehgoetz Martens K. A., Phillips J. R., Moustafa A. A., … Shine J. M. (2019). Supporting information for “Changes in structural network topology correlate with severity of hallucinatory behavior in Parkinson’s disease.” Network Neuroscience, 3(2), 521–538. 10.1162/netn_a_00078 PMC6444885 30984905

[bib35] Hall J. M., O’Callaghan C., Shine J. M., Muller A. J., Phillips J. R., Walton C. C., … Moustafa A. A. (2016). Dysfunction in attentional processing in patients with Parkinson’s disease and visual hallucinations. Journal of Neural Transmission (Vienna), 123(5), 503–507. 10.1007/s00702-016-1528-3 26940598

[bib36] Hepp D. H., Foncke E. M. J., Berendse H. W., Wassenaar T. M., Olde Dubbelink K. T. E., Groenewegen H. J., … Schoonheim M. M. (2017). Damaged fiber tracts of the nucleus basalis of Meynert in Parkinson’s disease patients with visual hallucinations. Scientific Reports, 7(1), 10112. 28860465 10.1038/s41598-017-10146-y PMC5579278

[bib37] Hepp D. H., Foncke E. M. J., Olde Dubbelink K. T. E., van de Berg W. D. J., Berendse H. W., & Schoonheim M. M. (2017). Loss of functional connectivity in patients with Parkinson disease and visual hallucinations. Radiology, 285(3), 896–903. 28952907 10.1148/radiol.2017170438

[bib38] Hoehn M. M., & Yahr M. D. (1998). Parkinsonism: Onset, progression, and mortality. Neurology, 50(2), 318–318. 9484345 10.1212/wnl.50.2.318

[bib39] Ibarretxe-Bilbao N., Ramírez-Ruiz B., Junqué C., Martí M. J., Valldeoriola F., Bargallo N., … Tolosa E. (2010). Differential progression of brain atrophy in Parkinson’s disease with and without visual hallucinations. Journal of Neurology, Neurosurgery, and Psychiatry, 81(6), 650–657. 10.1136/jnnp.2009.179655 19965847

[bib40] Intaite M., Noreika V., Soliunas A., & Falter C. M. (2013). Interaction of bottom-up and top-down processes in the perception of ambiguous figures. Vision Research, 89, 24–31. 23851264 10.1016/j.visres.2013.06.011

[bib41] Keri S., Decety J., Roland P. E., & Gulyas B. (2004). Feature uncertainty activates anterior cingulate cortex. Human Brain Mapping, 21(1), 26–33. 14689507 10.1002/hbm.10150 PMC6871971

[bib42] Lee J.-Y., Yoon E. J., Lee W. W., Kim Y. K., Lee J.-Y., & Jeon B. (2016). Lateral geniculate atrophy in Parkinson’s with visual hallucination: A trans-synaptic degeneration? Movement Disorders, 31(4), 547–554. 26846525 10.1002/mds.26533

[bib43] Leech R., & Sharp D. J. (2013). The role of the posterior cingulate cortex in cognition and disease. Brain, 137(1), 12–32. 23869106 10.1093/brain/awt162 PMC3891440

[bib44] Lenka A., Hegde S., Arumugham S. S., & Pal P. K. (2017). Pattern of cognitive impairment in patients with Parkinson’s disease and psychosis: A critical review. Parkinsonism and Related Disorders, 37, 11–18. 28057432 10.1016/j.parkreldis.2016.12.025

[bib45] Matsui H., Udaka F., Tamura A., Oda M., Kubori T., Nishinaka K., & Kameyama M. (2006). Impaired visual acuity as a risk factor for visual hallucinations in Parkinson’s disease. Journal of Geriatric Psychiatry and Neurology, 19(1), 36–40. 16449759 10.1177/0891988705284739

[bib46] Menon V., & Uddin L. Q. (2010). Saliency, switching, attention and control: A network model of insula function. Brain Structure and Function, 214(5–6), 655–667. 20512370 10.1007/s00429-010-0262-0 PMC2899886

[bib47] Meyer T., & Olson C. R. (2011). Statistical learning of visual transitions in monkey inferotemporal cortex. Proceedings of the National Academy of Sciences, 108(48), 19401–19406. 10.1073/pnas.1112895108 PMC3228439 22084090

[bib48] Misic B., Betzel R. F., Nematzadeh A., Goni J., Griffa A., Hagmann P., … Sporns O. (2015). Cooperative and competitive spreading dynamics on the human connectome. Neuron, 86(6), 1518–1529. 26087168 10.1016/j.neuron.2015.05.035

[bib49] Mori S., Crain B. J., Chacko V. P., & van Zijl P. C. (1999). Three-dimensional tracking of axonal projections in the brain by magnetic resonance imaging. Annals of Neurology, 45(2), 265–269. 9989633 10.1002/1531-8249(199902)45:2<265::aid-ana21>3.0.co;2-3

[bib50] Muller A. J., O’Callaghan C., Walton C. C., Shine J. M., & Lewis S. J. (2017). Retrospective neuropsychological profile of patients with Parkinson disease prior to developing visual hallucinations. Journal of Geriatric Psychiatry and Neurology, 30(2), 90–95. 28067106 10.1177/0891988716686830

[bib51] Muller A. J., Shine J. M., Halliday G. M., & Lewis S. J. (2014). Visual hallucinations in Parkinson’s disease: Theoretical models. Movement Disorders, 29(13), 1591–1598. 25154807 10.1002/mds.26004

[bib52] Nichols T. E., & Holmes A. P. (2002). Nonparametric permutation tests for functional neuroimaging: A primer with examples. Human Brain Mapping, 15(1), 1–25. 11747097 10.1002/hbm.1058 PMC6871862

[bib53] O’Callaghan C., Hall J. M., Tomassini A., Muller A. J., Walpola I. C., Moustafa A. A., … Lewis S. J. G. (2017). Visual hallucinations are characterized by impaired sensory evidence accumulation: Insights from hierarchical drift diffusion modeling in Parkinson’s disease. Biological Psychiatry: Cognitive Neuroscience and Neuroimaging, 2(8), 680–688. 29560902 10.1016/j.bpsc.2017.04.007

[bib54] Panichello M. F., Cheung O. S., & Bar M. (2012). Predictive feedback and conscious visual experience. Frontiers in Psychology, 3, 620 23346068 10.3389/fpsyg.2012.00620 PMC3549576

[bib55] Powers A. R., Kelley M., & Corlett P. R. (2016). Hallucinations as top-down effects on perception. Biological Psychiatry: Cognitive Neuroscience and Neuroimaging, 1(5), 393–400. 28626813 10.1016/j.bpsc.2016.04.003 PMC5469545

[bib56] Ramírez-Ruiz B., Junqué C., Martí M. J., Valldeoriola F., & Tolosa E. (2006). Neuropsychological deficits in Parkinson’s disease patients with visual hallucinations. Movement Disorders, 21(9), 1483–1487. 16705661 10.1002/mds.20965

[bib57] Ramírez-Ruiz B., Martí M. J., Tolosa E., Falcón C., Bargalló N., Valldeoriola F., & Junqué C. (2008). Brain response to complex visual stimuli in Parkinson’s patients with hallucinations: A functional magnetic resonance imaging study. Movement Disorders, 23(16), 2335–2343. 18785653 10.1002/mds.22258

[bib58] Rubinov M., & Sporns O. (2010). Complex network measures of brain connectivity: Uses and interpretations. NeuroImage, 52(3), 1059–1069. 19819337 10.1016/j.neuroimage.2009.10.003

[bib59] Sanchez-Ramos J. R., Ortollm R., & Paulson G. W. (1996). Visual hallucinations associated with Parkinson disease. Archives of Neurology, 53(23), 1265–1268. 8970453 10.1001/archneur.1996.00550120077019

[bib60] Schrag A. (2004). Psychiatric aspects of Parkinson’s disease: An update. Journal of Neurology, 251(7), 795–804. 15258780 10.1007/s00415-004-0483-3

[bib61] Shergill S. S., Walker Z., & Le Katona C. (1998). A preliminary investigation of laterality in Parkinson’s disease and susceptibility to psychosis. Journal of Neurology, Neurosurgery, and Psychiatry, 65(4), 610–611. 10.1136/jnnp.65.4.610 PMC2170290 9771806

[bib62] Shine J. M., Halliday G. H., Carlos M., Naismith S. L., & Lewis S. J. (2012). Investigating visual misperceptions in Parkinson’s disease: A novel behavioral paradigm. Movement Disorders, 27(4), 500–505. 22488861 10.1002/mds.24900

[bib63] Shine J. M., Halliday G. M., Gilat M., Matar E., Bolitho S. J., Carlos M., … Lewis S. J. (2014). The role of dysfunctional attentional control networks in visual misperceptions in Parkinson’s disease. Human Brain Mapping, 35(5), 2206–2219. 23760982 10.1002/hbm.22321 PMC6869072

[bib64] Shine J. M., Mills J. M. Z., Qiu J., O’Callaghan C., Terpening Z., Halliday G. M., … Lewis S. J. G. (2015). Validation of the psychosis and hallucinations questionnaire in non-demented patients with Parkinson’s disease. Movement Disorders Clinical Practice, 2(2), 175–181. 30363832 10.1002/mdc3.12139 PMC6183006

[bib65] Shine J. M., Muller A. J., O’Callaghan C., Hornberger M., Halliday G. M., & Lewis S. J. (2015). Abnormal connectivity between the default mode and the visual system underlies the manifestation of visual hallucinations in Parkinson’s disease: A task-based fMRI study. NPJ Parkinson’s Disease, 1, 15003 10.1038/npjparkd.2015.3 PMC5516559 28725679

[bib66] Shine J. M., O’Callaghan C., Halliday G. M., & Lewis S. J. (2014). Tricks of the mind: Visual hallucinations as disorders of attention. Progress in Neurobiology, 116, 58–65. 24525149 10.1016/j.pneurobio.2014.01.004

[bib67] Smith R. E., Tournier J. D., Calamante F., & Connelly A. (2012). Anatomically constrained tractography: Improved diffusion MRI streamlines tractography through effective use of anatomical information. NeuroImage, 62(3), 1924–1938. 22705374 10.1016/j.neuroimage.2012.06.005

[bib68] Sonuga-Barke E. J. S., & Castellanos F. X. (2007). Spontaneous attentional fluctuations in impaired states and pathological conditions: A neurobiological hypothesis. Neuroscience and Biobehavioral Reviews, 31(7), 977–986. 17445893 10.1016/j.neubiorev.2007.02.005

[bib69] Summerfield C., Egner T., Greene M., Koechlin E., Mangels J., & Hirsch J. (2006). Predictive codes for forthcoming perception in the frontal cortex. Science, 314(5803), 1311–1314. 17124325 10.1126/science.1132028

[bib70] Summerfield C., & Koechlin E. (2008). A neural representation of prior information during perceptual inference. Neuron, 59(2), 336–347. 18667160 10.1016/j.neuron.2008.05.021

[bib71] Sun Z., Wang F., Cui L., Breeze J., Du X., Wang X., … Zhang D. (2003). Abnormal anterior cingulum in patients with schizophrenia: A diffusion tensor imaging study. NeuroReport, 14(14), 1833–1836. 14534430 10.1097/00001756-200310060-00015

[bib72] Tombaugh T. N. (2004). Trail Making Test A and B: Normative data stratified by age and education. Archives of Clinical Neuropsychology, 19(2), 203–214. 15010086 10.1016/S0887-6177(03)00039-8

[bib73] Turner T. H., Cookson J. C., Wass J. A., Drury P. L., Price P. A., & Besser G. M. (1984). Psychotic reactions during treatment of pituitary tumours with dopamine agonists. British Medical Journal (Clinical Research Ed.), 289(6452), 1101–1103. 6435792 10.1136/bmj.289.6452.1101 PMC1443261

[bib74] van den Heuvel M. P., & Sporns O. (2011). Rich-club organization of the human connectome. Journal of Neuroscience, 31(44), 15775–15786. 22049421 10.1523/JNEUROSCI.3539-11.2011 PMC6623027

[bib75] Verstraete E., van den Heuvel M. P., Veldink J. H., Blanken N., Mandl R. C., Hulshoff Pol H. E., & van den Berg L. H. (2010). Motor network degeneration in amyotrophic lateral sclerosis: A structural and functional connectivity study. PLoS ONE, 5(10), e13664 21060689 10.1371/journal.pone.0013664 PMC2965124

[bib76] Weissman D. H., Roberts K., Visscher K., & Woldorff M. (2006). The neural bases of momentary lapses in attention. Nature Neuroscience, 9(7), 971. 16767087 10.1038/nn1727

[bib77] Xia M., Wang J., & He Y. (2013). BrainNet Viewer: A network visualization tool for human brain connectomics. PLoS One, 8(7), e68910 23861951 10.1371/journal.pone.0068910 PMC3701683

[bib78] Yao N., Shek-Kwan Chang R., Cheung C., Pang S., Lau K. K., Suckling J., … Chua S. E. (2014). The default mode network is disrupted in Parkinson’s disease with visual hallucinations. Human Brain Mapping, 35(11), 5658–5666. 24985056 10.1002/hbm.22577 PMC4657500

[bib79] Yeo B. T., Krienen F. M., Sepulcre J., Sabuncu M. R., Lashkari D., Hollinshead M., … Buckner R. L. (2011). The organization of the human cerebral cortex estimated by intrinsic functional connectivity. Journal of Neurophysiology, 106(3), 1125–1165. 21653723 10.1152/jn.00338.2011 PMC3174820

[bib80] Zalesky A., Fornito A., & Bullmore E. T. (2010). Network-based statistic: Identifying differences in brain networks. NeuroImage, 53(4), 1197–1207. 20600983 10.1016/j.neuroimage.2010.06.041

[bib81] Zalesky A., Fornito A., Cocchi L., Gollo L. L., van den Heuvel M. P., & Breakspear M. (2016). Connectome sensitivity or specificity: Which is more important? NeuroImage, 142, 407–420. 27364472 10.1016/j.neuroimage.2016.06.035

