# Supplementary material for: Changes in structural network topology correlate with severity of hallucinatory behavior in Parkinson’s disease
Source: Netw Neurosci. 2019 Mar 1;3(2):521–38. doi: 10.1162/netn_a_00078 (PMC6444885; doi:10.1162/netn_a_00078)
Supplement: Supplementary file 1 [file netn-03-521-s001.pdf]

Hall, J. M., O'Callaghan, C., Muller, A. J., Ehgoetz Martens, K. A., Phillips, J. R., Moustafa, A. A., Lewis, S. J. G., & Shine, J. M. (2019). Supporting information for "Changes in structural network topology correlate with severity of hallucinatory behavior in Parkinson's disease." *Network Neuroscience*, *3*(2), 521–538. https://doi.org/10.1162/netn_a_00078

Supplementary Materials

Supplementary Table 1. Nodes of the diverse club

Node	Participation coefficient	Inclusion sub-network
'Left-Putamen'	0.814	✓
'Right-Pallidum'	0.811	✓
'Right-Putamen'	0.808	✓
'Left-Pallidum'	0.807	✓
'Right-Thalamus-Proper'	0.805	✓
'Right-Caudate'	0.768	✓
'ctx-rh-precuneus_4'	0.734	✓
'ctx-lh-superiorparietal_5'	0.731	✓
'ctx-lh-superiorparietal_4'	0.724	✓
'ctx-rh-superiorfrontal_5'	0.721	✓
'ctx-rh-isthmuscingulate_1'	0.716	✓
'Left-Hippocampus'	0.713	✓
'ctx-lh-insula_3'	0.705	✓
'ctx-rh-rostralmiddlefrontal_5'	0.695	✓
'ctx-lh-superiorfrontal_3'	0.664	✓
'ctx-rh-superiorparietal_3'	0.658	✓
'ctx-lh-lateraloccipital_1'	0.653	✓
'ctx-rh-postcentral_5'	0.612	-

ctx-lh = left hemisphere; ctx-rh = right hemisphere

Psychosis and Hallucinations Questionnaire, part A

PSYCHOSIS AND HALLUCINATIONS QUESTIONNAIRE

Appendix

The self-administered 'Psychosis and Hallucinations Questionnaire'

Psychosis and Hallucinations Questionnaire (PsycH-Q)

Please complete the following 20 questions relating to changes in vision, thinking and sleep that can sometimes occur in the normal progression of Parkinson's disease.

If the symptom was present in the past month, please rate how frequently the symptom occurred and how distressing you found it as None, Mild, Moderate, or Severe.

None = not at all distressing
Mild = somewhat distressing
Moderate = very distressing
Severe = extremely distressing

Over the past month, how often did you:

If you experienced the symptom, how distressing was it for you?

1. Feel like someone is in the room with you that's not really there?

   Never ⓪ — **<1 time per week** ① — **Weekly** ② — **Most days a week** ③ — **Daily** ④
   None [1] — **Mild** [2] — **Moderate** [3] — **Severe** [4]

2. Feel like something is lurking in the corner of your vision?

   Never ⓪ — **<1 time per week** ① — **Weekly** ② — **Most days a week** ③ — **Daily** ④
   None [1] — **Mild** [2] — **Moderate** [3] — **Severe** [4]

3. Feel that something is passing by you that you can't make out?

   Never ⓪ — **<1 time per week** ① — **Weekly** ② — **Most days a week** ③ — **Daily** ④
   None [1] — **Mild** [2] — **Moderate** [3] — **Severe** [4]

4. See shadowy forms that aren't really there?

   Never ⓪ — **<1 time per week** ① — **Weekly** ② — **Most days a week** ③ — **Daily** ④
   None [1] — **Mild** [2] — **Moderate** [3] — **Severe** [4]

5. Mistake an object for something else e.g. a snake instead of a hose?

   Never ⓪ — **<1 time per week** ① — **Weekly** ② — **Most days a week** ③ — **Daily** ④
   None [1] — **Mild** [2] — **Moderate** [3] — **Severe** [4]

6. See people or things (e.g. animals) that aren't there?

   Never ⓪ — **<1 time per week** ① — **Weekly** ② — **Most days a week** ③ — **Daily** ④
   None [1] — **Mild** [2] — **Moderate** [3] — **Severe** [4]

7. Hear voices or things that aren't real?

   Never ⓪ — **<1 time per week** ① — **Weekly** ② — **Most days a week** ③ — **Daily** ④
   None [1] — **Mild** [2] — **Moderate** [3] — **Severe** [4]

Over the past month, how often did you:

If you experienced the symptom, how distressing was it for you?

8. Touch or feel things touching you which aren't real?

Never	<1 time per week	Weekly	Most days a week	Daily	None	Mild	Moderate	Severe
0	1	2	3	4	1	2	3	4

9. Smell odours which aren't real?

Never	<1 time per week	Weekly	Most days a week	Daily	None	Mild	Moderate	Severe
0	1	2	3	4	1	2	3	4

10. Taste things which are not there?

Never	<1 time per week	Weekly	Most days a week	Daily	None	Mild	Moderate	Severe
0	1	2	3	4	1	2	3	4

If you experienced any of the symptoms from 1 to 10,

	Yes	No
Did you experience any of the symptoms only when you were about to fall asleep?	1	0
Did you think that these experiences could be real?	1	0
If **Yes**, could you be convinced otherwise?	1	0
Did these experiences frighten you?	1	0
Have you ever experienced these symptoms outside of the past month?	1	0

Over the past month, how often did you:

If you experienced the symptom, how distressing was it for you?

11. Think people are acting against you? e.g. stealing, hiding things

Never	<1 time per week	Weekly	Most days a week	Daily	None	Mild	Moderate	Severe
0	1	2	3	4	1	2	3	4

12. Feel threatened by the people around you?

Never	<1 time per week	Weekly	Most days a week	Daily	None	Mild	Moderate	Severe
0	1	2	3	4	1	2	3	4

13. Think people are trying to trick you? e.g. pretending to be other people

Never	<1 time per week	Weekly	Most days a week	Daily	None	Mild	Moderate	Severe
0	1	2	3	4	1	2	3	4

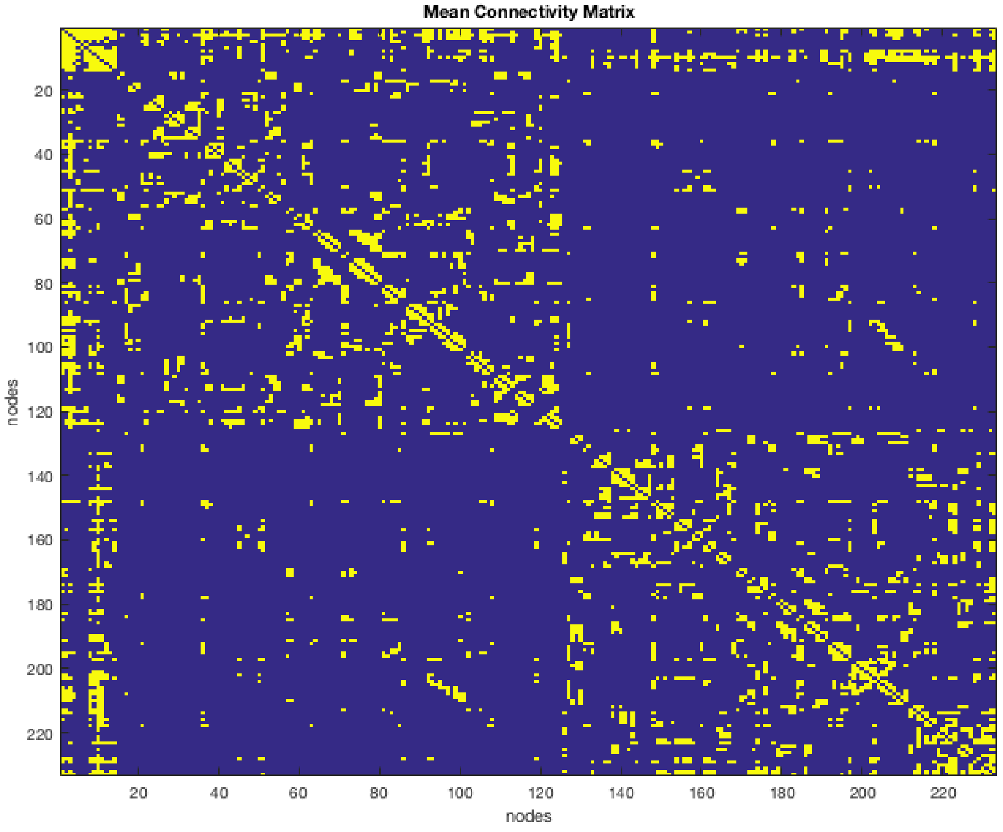

Supplementary figure 1. Mean connectivity matrix (thresholded)
Connectivity matrix is deposited at github.com/juliemaehall

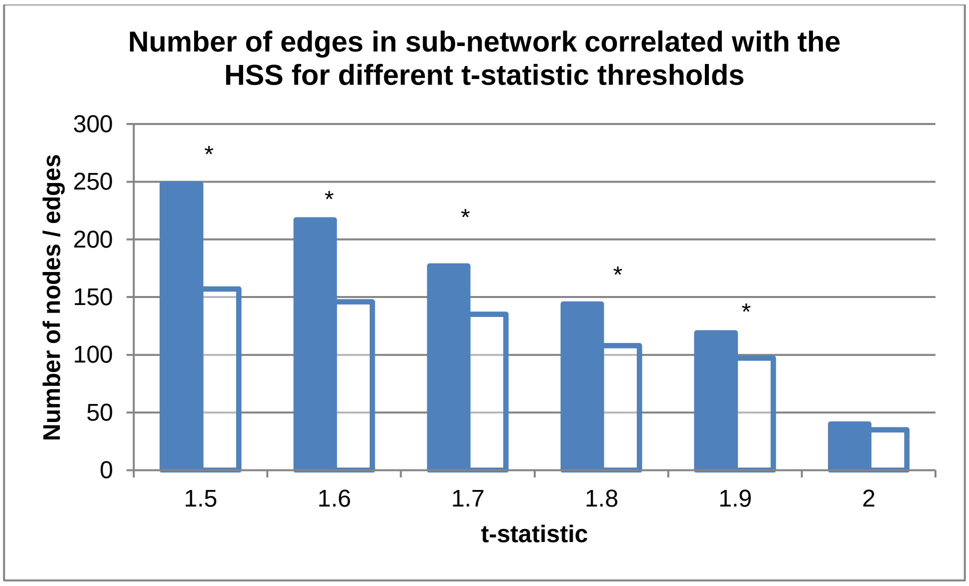

Supplementary figure 2. Size of sub-networks correlated with the HSS for different t-statistics. Blue bars represent the number of edges; white bars represent the number of nodes. Asterisks (*) represent a significant correlation ($p < 0.05$).
